# Distinct Vulnerability and Resilience of Human Neuroprogenitor Subtypes in Cerebral Organoid Model of Prenatal Hypoxic Injury

**DOI:** 10.3389/fncel.2019.00336

**Published:** 2019-07-30

**Authors:** Nicolas Daviaud, Clément Chevalier, Roland H. Friedel, Hongyan Zou

**Affiliations:** ^1^Nash Family Department of Neuroscience, Friedman Brain Institute, New York, NY, United States; ^2^The Center for Microscopy and Molecular Imaging, Université Libre de Bruxelles, Brussels, Belgium; ^3^Department of Neurosurgery, Icahn School of Medicine at Mount Sinai, New York, NY, United States

**Keywords:** cerebral organoids, prenatal hypoxic injury, human corticogenesis, neural stem cell, neuroprogenitor, outer radial glia

## Abstract

Prenatal hypoxic injury (HI) is a leading cause of neurological disability. The immediate and long-term effects of hypoxia on progenitor homeostasis and developmental progression during early human brain development remain unclear. This gap is due to difficulty to access human fetal brain tissues and inadequate animal models to study human corticogenesis. Recent optimizations of cerebral organoid models derived from human embryonic stem (ES) cells present new opportunities to investigate pathophysiology of prenatal HI. Here, we implemented a transient HI model using human cerebral organoids with dorsal forebrain specification. We demonstrated that transient hypoxia resulted in immediate and prolonged apoptosis in cerebral organoids, with outer radial glia (oRG), a progenitor population more prominent in primates, and differentiating neuroblasts/immature neurons suffering larger losses. In contrast, neural stem cells in ventricular zone displayed relative resilience to HI and exhibited a shift of cleavage plane angle favoring symmetric division, thereby providing a mechanism to replenish the stem cell pool. Furthermore, we defined the vulnerable window and neurodifferentiation stages that are particularly sensitive to HI. Understanding cell type-specific and stage-dependent effects of prenatal HI on survival and mitotic behavior of human neuroprogenitor subtypes during early human corticogenesis helps elucidate the etiology of neurodevelopmental disorders, and provides a therapeutic starting point to protect the vulnerable populations at critical timeframes.

## Introduction

Prenatal hypoxic injury (HI) is a leading cause of neurological disability, with an incidence of 2–4 per 1000 live births (Badr Zahr and Purdy, 2006; Glass and Ferriero, 2007). Deprivation of oxygen due to placental insufficiency, umbilical cord occlusion, premature birth, or obstetric complications can result in immediate cellular damages but also long-term dysfunction of neuroprogenitor cells (NPCs), leading to developmental anomaly and increased risk of neurological disorders later in life (Schump, 2018). Even a short, transient hypoxic episode, e.g., 30 min, can trigger fetal brain injury at mid-gestation (Mallard et al., 1993; Gunn and Bennet, 2009).

Hypoxic conditions alter brain development as a result of increased apoptosis and a delay in maturation (Salmaso et al., 2014). However, the immediate and long-term effects of hypoxia on NPC homeostasis and differentiation trajectory during early human brain development remain poorly understood, and this is due to relative inaccessibility of human fetal brain tissues and inadequate animal models to study human corticogenesis.

Recent advances in 3D cerebral organoid cultures derived from human embryonic stem cells (hESC) or induced pluripotent stem cells (iPSC) (Kadoshima et al., 2013; Lancaster et al., 2013; Lancaster and Knoblich, 2014; Camp et al., 2015) provide new avenues to implement reproducible models to study cell type- and stage-specific effects of HI on early human cortical development. Cerebral organoid cultures take advantage of the enormous self-organizing potential of neural stem cells (NSCs) and differentiating NPCs to develop into complex structures that mimic early to mid-gestation human brain development (Camp et al., 2015). Cerebral organoids can be differentiated toward dorsal forebrain specification containing ventricle-like structures aligned with ventricular zone (VZ)-like germinal regions populated by NSCs, subventricular zone (SVZ) populated by committed progenitors in various stages of differentiation and migration, and a rudimentary cortical plate (CP) occupied by cortical neurons in a stratified layout (Lancaster et al., 2013). In addition, human cerebral organoids contain a progenitor domain that is more prominent in primates, termed outer SVZ (oSVZ), which is populated by outer radial glia (oRG), which support the evolutionary expansion of human neocortex (Hansen et al., 2010; Dehay et al., 2015; Pollen et al., 2015).

Here, we implemented an organoid-based platform to model prenatal transient HI during early human brain development. As compared to 2D adherent cultures, cerebral organoids contain different neuroprogenitor subtypes that are maintained in a complex 3D cytoarchitecture with stereotypical spatial alignment and proper cell-cell interactions, thus better recapitulating cellular diversity in distinct niches as in developing human brains. The organoid model allows us to address the following fundamental questions not possible with conventional models: (i) relative vulnerability of human neuroprogenitor subtypes to HI, (ii) stability of NSC reserve and potential compensatory mechanisms, and (iii) vulnerable window and differentiation stages that are most affected by HI.

Using the cerebral organoid model, we found that transient HI resulted in immediate and prolonged apoptosis, with FAM107^+^ oRG progenitors and Doublecortin^+^ (DCX^+^) neuroblasts/immature neurons suffering larger losses. TBR2^+^ intermediate progenitors (IP) also suffer losses but only at later stages. SOX2^+^ NSCs residing in the VZ-like region remained stable, attributable to intrinsic resilience and a shift of cell division mode for self-expansion. Consistently, timed EdU and BrdU pulse-chase studies of isochronic progenitor cohorts revealed that the differentiating population in SVZ/CP appeared more affected by HI than active cycling NSCs in VZ. Pasca et al. (2019) have recently also reported a transient HI model based on human cortical spheroids (hCS) derived from multiple iPSC lines, which similarly demonstrated particular vulnerability of TBR2^+^ IP to transient HI.

Taken together, human cerebral organoids can be used to implement a reproducible HI paradigm to dissect the etiology of cortical dysgenesis associated with hypoxic insults. Our study and the study of Pasca et al. (2019) demonstrated the broad utility of cerebral organoids for modeling pathophysiology of human neurodevelopmental disorders.

## Materials and Methods

### Cerebral Organoid Generation

Human cerebral organoids were generated from H9 hESCs as described (Lancaster et al., 2013; Lancaster and Knoblich, 2014; Daviaud et al., 2018), with minor modifications. In brief, H9 hESCs were plated in round bottom ultra-low attachment 96-well plates to form embryoid bodies, followed by neural induction. After neuroepithelium emerged, organoids were embedded in Matrigel droplets and cultured in 6 cm Petri dishes for 4 days and were then placed on an orbital shaker at 85 rpm until analyses.

### Hypoxia Injury Induction in Cerebral Organoids

Twenty-eight days after differentiation from hESCs, organoids were cultured in a hypoxic chamber (3% O_2_, 5% CO_2_) (BioSpherix) for 24 h, and then switched back to normoxic condition (21% O_2_, 5% CO_2_) until analysis.

### Labeling of Isochronic NPC Cohorts

Organoids were pulsed with 15 μM of 5-bromo-2′-deoxyuridine (BrdU, Acros Organics) or 10 μM 5-ethynyl-2′-deoxyuridine (EdU, Thermo Fisher) for 30 min. Label-retaining cells (LRCs) were revealed by EdU Click-iT kit (Thermo Fisher) or anti-BrdU antibody (rat, Thermo Scientific MA182718, 1:400).

### Quantification and Statistical Analysis

All quantitative analyses were carried out in 100 μm (width) × 300 μm (height) radial columns spanning all cortical layers near organoid surface regions as shown in Figure 2A, unless otherwise specified. At least 2–3 different organoids from three independent batches were used for each condition, and 1–3 representative images from each organoid were quantified (Fiji software) (Schindelin et al., 2012). Bar graphs are presented as mean with standard error of the mean (SEM). Box plots represent data extending from 25th to 75th percentiles, with whisker depicting median and min to max (GraphPad Prism 7.0).

For individual comparison between two conditions, normality of data was assessed using a Shapiro–Wilk test followed by a Student *t*-test. Differences between multiple conditions were determined using a two-way analysis of variance (ANOVA) test, followed by a Tukey *post hoc* test. To analyze frequencies distribution, a Chi-square test was performed (see Supplementary Materials for details).

## Results

### Establishment and Characterization of HI Model With Human Cerebral Organoids

**FIGURE 1:**
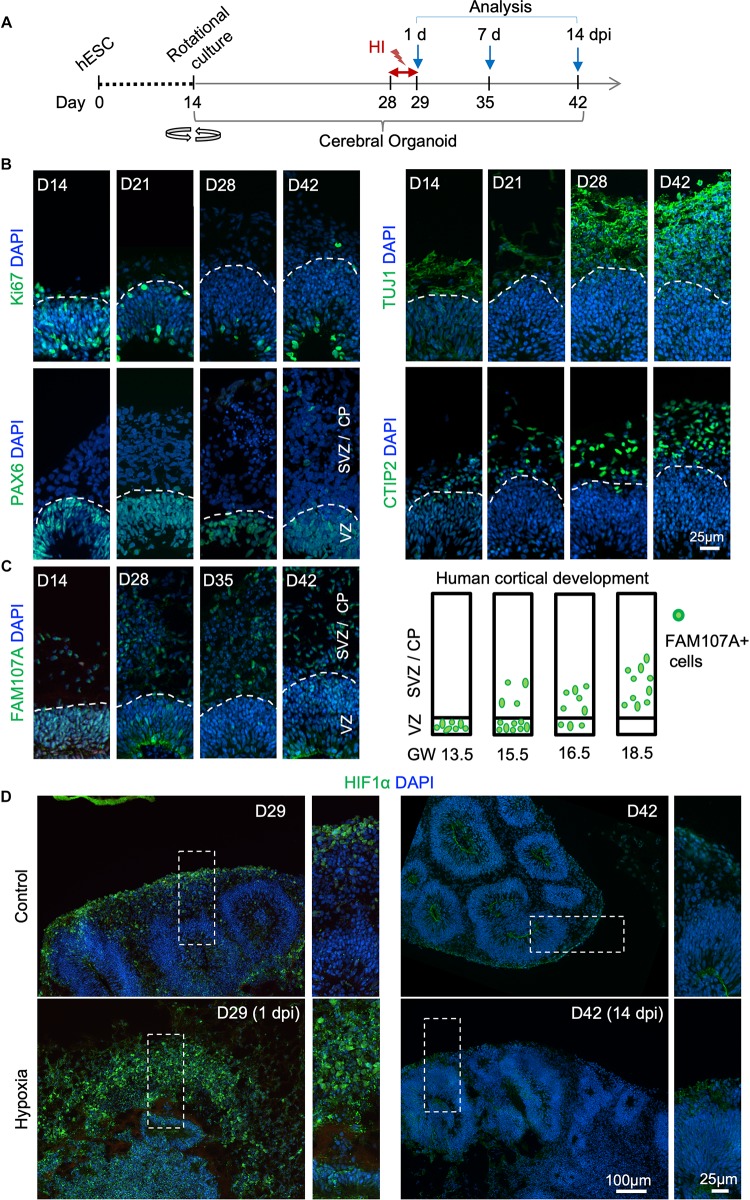
Transient hypoxia model in human cerebral organoids. **(A)** Schematic diagram of transient hypoxic injury (HI) model with hESC-derived cerebral organoids. D0 represents the starting day of differentiation, D14 denotes the time when organoids were transferred to culture on orbital shaker. At D28, organoids were subjected to 3% O_2_ in a hypoxic chamber for 24 h, followed by return to 21% O_2_ culture condition for the remaining culture periods until analysis. **(B)** IF images for the indicated markers in cerebral organoids demonstrate progressive development of cortex-like structures and appearance of immature neurons (TUJ1^+^) and deep-layer cortical neurons (CTIP2^+^) from D14–D42. Proliferating (Ki67^+^) NSCs (PAX6^+^) reside in VZ-like germinal region, and offspring in various stages of differentiation migrate out of VZ to populate outer layers, designated as SVZ/CP-like structures. White dashed lines delineate border between VZ and SVZ/CP. Note higher cellular density in the VZ as visualized by DAPI nuclear counterstaining. **(C)** Left, IF images demonstrate progressive development of oRGs that express FAM107A. Right, schematic depiction of spatial localization of FAM107A^+^ oRGs during human brain development at the indicated gestational week (GW), adapted from Pollen et al. (2015). **(D)** Representative IF images show stabilization of HIF-1α at D29 (1 dpi) after hypoxia episode as compared to control, while only baseline HIF-1α immunofluorescence was detected at D42 (14 dpi). High magnification images of boxed areas are shown on the right.

To develop a reproducible experimental paradigm to model prenatal hypoxia during early human brain development, we adapted the protocol of Lancaster and colleagues with minor modifications to derive consistent cerebral organoids from human ESCs with dorsal forebrain specification (Supplementary Figure S1A; Lancaster et al., 2013; Lancaster and Knoblich, 2014). Human ESCs were first differentiated into embryoid bodies, followed by neuroectodermal differentiation. Tissues were then embedded in Matrigel droplets and cultured first in stationary and then rotating condition on an orbital shaker (Supplementary Figure S1A). Over the next 4 weeks of culture, we observed rapid maturation of cerebral organoids with appearance of ventricle-like structures aligned with a VZ-like proliferative zone and a rudimental CP, separated by an SVZ-like transitional zone (Figures 1B,C and Supplementary Figure S1B). Specifically, at D14 after start of differentiation, when culture on orbital shaker started, abundant proliferating cells (Ki67^+^) expressing radial glia (RG) marker PAX6 were detected in neuroepithelium (Figure 1B). From D21 to D42, a majority of Ki67^+^ cells remained in the VZ, while a small fraction of Ki67^+^ cells appeared in outer layers representing intermediate progenitors expressing TBR2 (EOMES) and neuroblasts expressing DCX (Hevner et al., 2006; Figure 1B and Supplementary Figure S1B). During the same period, CP steadily increased in thickness with progressively more cells expressing immature neuronal marker β-III tubulin (TUJ1) and deep layer subcortical projection neuron marker CTIP2 (Molyneaux et al., 2005; Figure 1B and Supplementary Figure S1B).

Human cerebral organoids also contain a progenitor domain that is much more prominent in primates, populated with oRGs expressing FAM107A (Lancaster et al., 2013; Pollen et al., 2015). It has been shown that the localization of FAM107A^+^ oRGs during corticogenesis reflects the fetal gestational week (GW) during human embryogenesis (Pollen et al., 2015). Indeed, we found a progressive change of localization of FAM107A^+^ cells in organoids, from mainly VZ at D14 to mainly outer layers at D42 (Figure 1C and Supplementary Figure S1B). Based on this correlation, we estimated that our D28–D42 cerebral organoids recapitulated the developmental stage of human fetal cortex at early midgestation (∼GW14.5–18.5).

We next adapted a hypoxia protocol used widely in brain slice cultures for human cerebral organoids (Morales et al., 2007; Wise-Faberowski et al., 2009). We first tested extended exposure of cerebral organoids to 3% oxygen tension for 14 days (D28–D42), which resulted in poorly developed organoids, massive cell death, and cytoarchitectural collapse (Supplementary Figures S2A,B). The VZ and cortical layers were highly compromised with markedly diminished NSC pool (SOX2^+^ or PAX6^+^) and near obliteration of neuronal populations (β-III tubulin^+^) (Supplementary Figure S2C).

To model milder effects of non-lethal prenatal hypoxia frequently encountered in human newborns, we tested a transient hypoxia paradigm wherein cerebral organoids were subjected to 3% O_2_ for 24 h from D28–D29, and then returned to normoxia for the remaining culture period (Figure 1A). We reasoned that such a transient HI paradigm would preserve overall structural integrity of cerebral organoids without massive cell death and cytoarchitectural collapse, allowing us to study subtle changes from non-lethal HI during early human corticogenesis rather than irreversible cellular damages. Indeed, after transient HI, we found no overt structural alterations in ventricle size or layered organization of VZ and SVZ/CP at D42, i.e., 14-day post initiation of HI (dpi) (Supplementary Figure S3A). Immunostaining for radial glia marker SOX2 and immature neuronal marker β-III tubulin also revealed stereotypical organization of germinal zone and cortical layer with no cellular ectopia (Supplementary Figure S3B). Similarly, measurement of thickness of cortical columns as a whole, or VZ and SVZ/CP layers separately, indicated no significant differences between HI and control conditions (Supplementary Figure S3C).

Hypoxia-inducible factor (HIF) is a protein complex that senses oxygen tension through stabilization of the complex in hypoxic conditions (Maxwell et al., 1999). Immediately after transient hypoxia, we detected an increase of HIF-1α immunostaining in organoids at D29, with cells in outer layers exhibiting higher levels than cells in VZ (Figure 1D). Notably, by D42 (14 dpi), HIF-1α levels had returned to baseline with no detectable differences between HI and control conditions (Figure 1D). We continued our studies with the transient HI paradigm to model non-lethal prenatal hypoxia corresponding to early human corticogenesis.

### Prolonged Apoptosis in Cerebral Organoids After Transient Hypoxia

**FIGURE 2:**
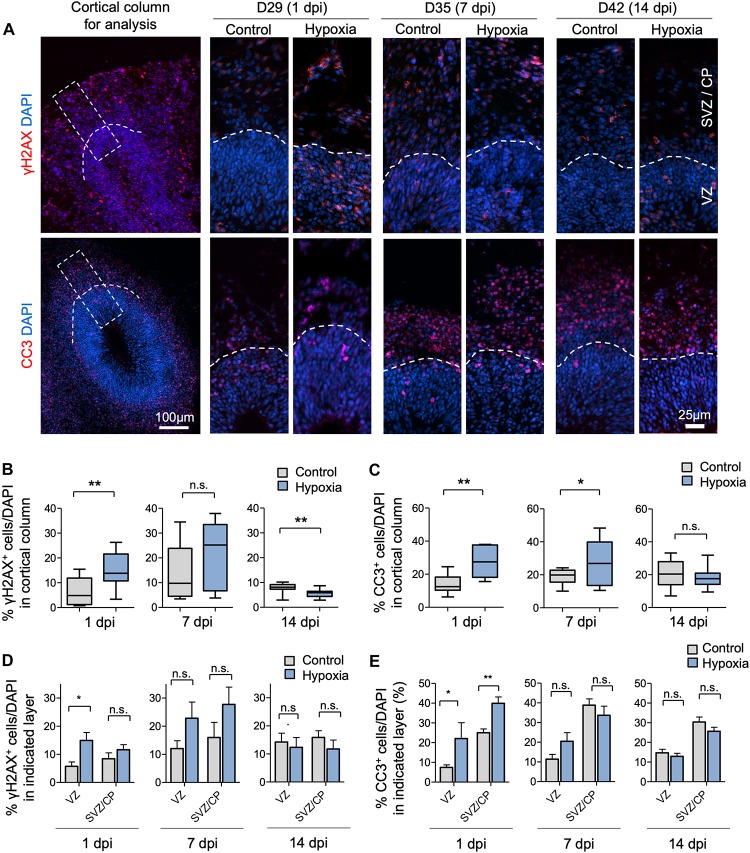
Transient hypoxia induces immediate DNA damage and prolonged apoptosis in cerebral organoids. **(A)** Representative IF images for γH2AX and cleaved caspase 3 (CC3) in cerebral organoids. Boxed area in lower magnification images in left panel denotes the analyzed area, i.e., a 100 μm wide × 300 μm high radial column spanning all cortical layers (VZ, SVZ, and CP). Corresponding cortical areas of the same size were used for all quantifications in our studies to ensure consistency. Higher magnification images are shown in right panels. White dashed lines demarcate VZ and SVZ/CP. **(B,C)** Quantifications show the percentage of DAPI^+^ cells that are positive for γH2AX **(B)** or CC3 **(C)**. Analyses were carried out in 100 μm × 300 μm cortical column as shown in **(A)**. Student’s *t* test, *n* = 9 independent organoids from 3 different batches. **(D,E)** Quantifications of percentage of DAPI^+^ cells that are positive for γH2AX **(D)** or CC3 **(E)** in VZ or outer layers (SVZ/CP). Two-way ANOVA followed by a Tukey *post hoc* test; *n* = 9. ^*^p < 0.05; ^∗∗^*p* < 0.01; n.s., not significant.

To assess the effects of transient HI on DNA integrity and cell survival in cerebral organoids, we performed immunofluorescence (IF) staining for γH2AX and cleaved caspase 3 (CC3), markers for DNA double-strand break and apoptosis, respectively. We observed an increase in the prevalence of γH2AX^+^ cells immediately after HI at D29 (6% in control vs. 15% after HI, *p* < 0.001) (Figures 2A,B). The prevalence of γH2AX^+^ cells only showed a trend of increase by 7 dpi at D35 (14% in control vs. 21% after HI, *p* = 0.23), but had returned to basal levels with even a modest reduction by 14 dpi at D42 (8% in control vs. 6% after HI, *p* = 0.005) (Figures 2A,B), perhaps reflecting enhanced DNA repair capability among the surviving population. In parallel, transient HI resulted in a significant increase in apoptotic rates at 1 dpi (14% in control vs. 28% after HI, *p* = 0.002) and 7 dpi (19% in control vs. 27% after HI, *p* = 0.002), but no longer at 14 dpi (20% in control vs. 18% after HI, *p* = 0.25) (Figures 2A,C).

To examine if DNA damage or apoptosis occurred predominantly in neural precursors or differentiating populations, we quantified the prevalence of γH2AX^+^ and CC3^+^ cells in VZ and outer layers (SVZ/CP) separately. At 1 dpi, we detected a marked increase in the percentage of γH2AX^+^ cells in VZ (6% in control vs. 15% after HI, *p* < 0.01), but no significant difference in SVZ/CP, consistent with higher replication stress in the proliferative region. However, by 7 and 14 dpi, no significant differences in the prevalence of γH2AX^+^ cells were detected in either VZ or SVZ/CP between control and hypoxia conditions (Figure 2D). Similarly, transient hypoxia induced an increase in apoptotic rates in both VZ and SVZ/CP at 1 dpi, but no significant differences at 7 and 14 dpi (Figure 2E). It is worth noting that throughout maturation of cerebral organoids in both control and hypoxia conditions, CC3^+^ cells were localized predominantly in SVZ/CP relative to VZ (∼25% vs. ∼7%, respectively, *p* < 0.001) despite higher replication stress in cells in VZ (Figures 2D,E). These results indicated that neural precursors in the VZ might be endowed with better intrinsic capability to cope with DNA damage or hypoxic metabolic stress than their differentiating progenies in SVZ/CP.

### Outer Radial Glia and Neuroblasts Display Higher Sensitivity to HI

**FIGURE 3:**
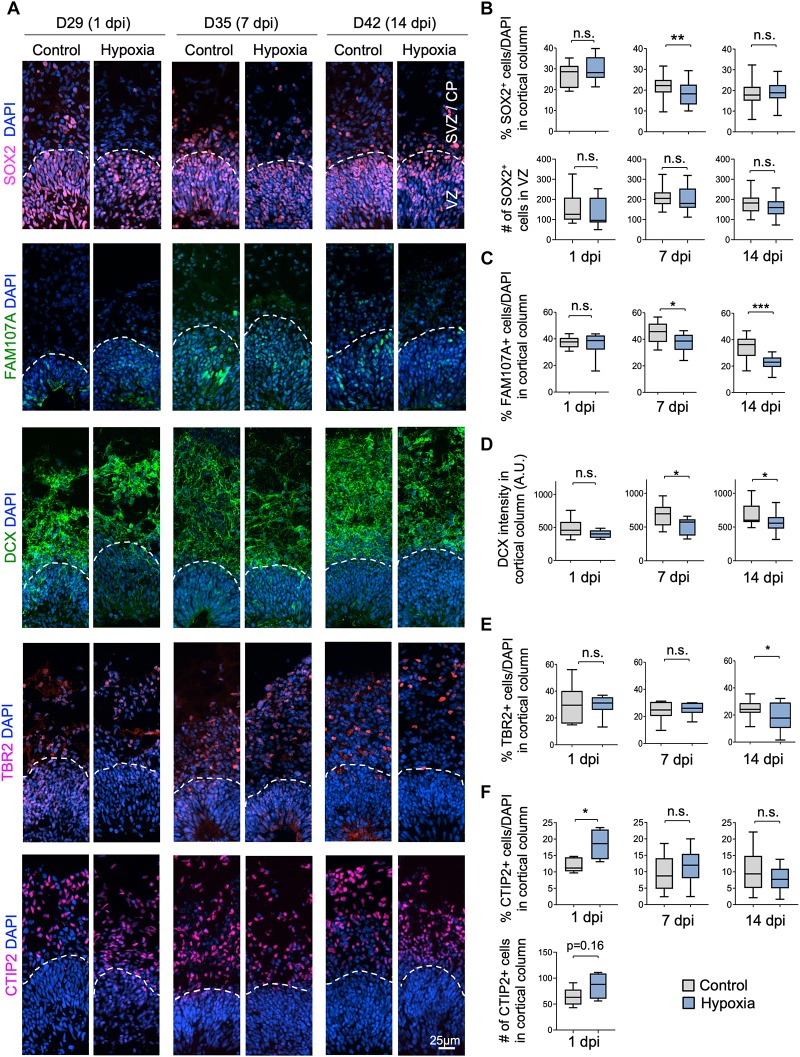
Cellular distinction of different neural populations in response to HI. **(A)** IF images of representative 100 × 300 μm cortical columns labeled for the indicated markers at the indicated time points after hypoxia or control. White dashed lines demarcate VZ and outer layers (SVZ/CP). **(B)** Top graphs: quantifications of the percentage of DAPI^+^ cells across all cortical layers in radial columns that are positive for SOX2 at the indicated time points after hypoxia or control. Bottom graphs: quantifications of the number of SOX2^+^ cells in VZ-like layer at the indicated time points. Student’s *t* test, *n* = 9 organoids from three different batches. **(C)** Quantifications of the percentage of DAPI^+^ cells in cortical columns that are positive for FAM107A. Student’s *t* test, *n* = 9. **(D)** Quantifications of immunostaining intensity for DCX normalized to unit area (expressed as A.U.). Student’s *t* test, *n* = 9. **(E)** Quantifications of the percentage of DAPI^+^ cells across all cortical layers in radial columns that are positive for TBR2 at the indicated time points after hypoxia or control. **(F)** Top: quantifications of percentage of DAPI^+^ cells positive for CTIP2 in radial columns. Bottom: quantifications of the number of CTIP2^+^ cell in radial columns at 1 dpi. Student’s *t* test, *n* = 9. A.U., arbitrary unit. ^*^p < 0.05; ^∗∗^*p* < 0.01; ^∗∗∗^*p* < 0.001; n.s., not-statistically significant.

We next investigated if specific neural cell types might be particularly vulnerable to transient hypoxia. To this end, we compared abundance of different neural populations in control vs. hypoxia conditions at 1, 7, or 14 dpi. Immediately after HI, we found no significant difference in the prevalence of SOX2^+^ cells, but at 7 dpi, there was a small (∼3%) decrease of the percent of SOX2^+^ cells after HI relative to control (*p* < 0.01), and by 14 dpi, the number became comparable between conditions (Figures 3A,B). Since a majority of SOX2^+^ cells resided in VZ, but a small proportion were localized in outer layers, representing oRGs (Pollen et al., 2015; Thomsen et al., 2015), we further quantified the number of SOX2^+^ cells only in VZ, which showed no significant differences between control and HI conditions at all time points. These data revealed stability of the progenitor pool in VZ throughout recovery period after HI, but also suggested that SOX2^+^ oRG population in oSVZ might be particularly vulnerable to HI.

Indeed, we observed a reduction of the prevalence of FAM107^+^ cells in cerebral organoids at 7 dpi (45% in control vs. 37% after HI, *p* < 0.05) and at 14 dpi (34% in control vs. 22% after HI, *p* < 0.001), but no immediate drop at 1 dpi (Figures 3A,C). Similarly, DCX staining intensity displayed a significant drop at 7 and 14 dpi relative to controls, but not at 1 dpi (Figures 3A,D). Likewise, we observed a significant reduction of the percent of TBR2^+^ IPs in the cortical columns at 14 dpi (25% in control vs. 18% after HI, *p* = 0.02), but no significant differences at 1 and 7 dpi (Figures 3A,E).

In comparison, the prevalence of CTIP2^+^ neurons showed no significant change between conditions at 7 and 14 dpi, but surprisingly an increase at 1 day post HI (Figures 3A,F). We suspected that this might stem from a demise of other cell types at 1 dpi when massive cell death occurred in outer layers (∼40% in SVZ/CP, Figure 2C). Indeed, when we quantified the absolute number of CTIP2^+^ cells in radial cortical columns, the difference between HI and control conditions was not statistically significant (Figure 3F). Taken together, different neural populations display different sensitivity and resilience to HI, with FAM107A^+^ oRGs, DCX^+^ neuroblasts/immature neurons and TBR2^+^ IPs being more vulnerable even after an extended recovery period. By comparison, SOX2^+^ NSCs in VZ and CTIP2^+^ deep-layer subcortical projection neurons showed relative resilience to HI.

### Transient Hypoxia Induces a Shift of Cleavage Plane of Neural Precursors in VZ

**FIGURE 4:**
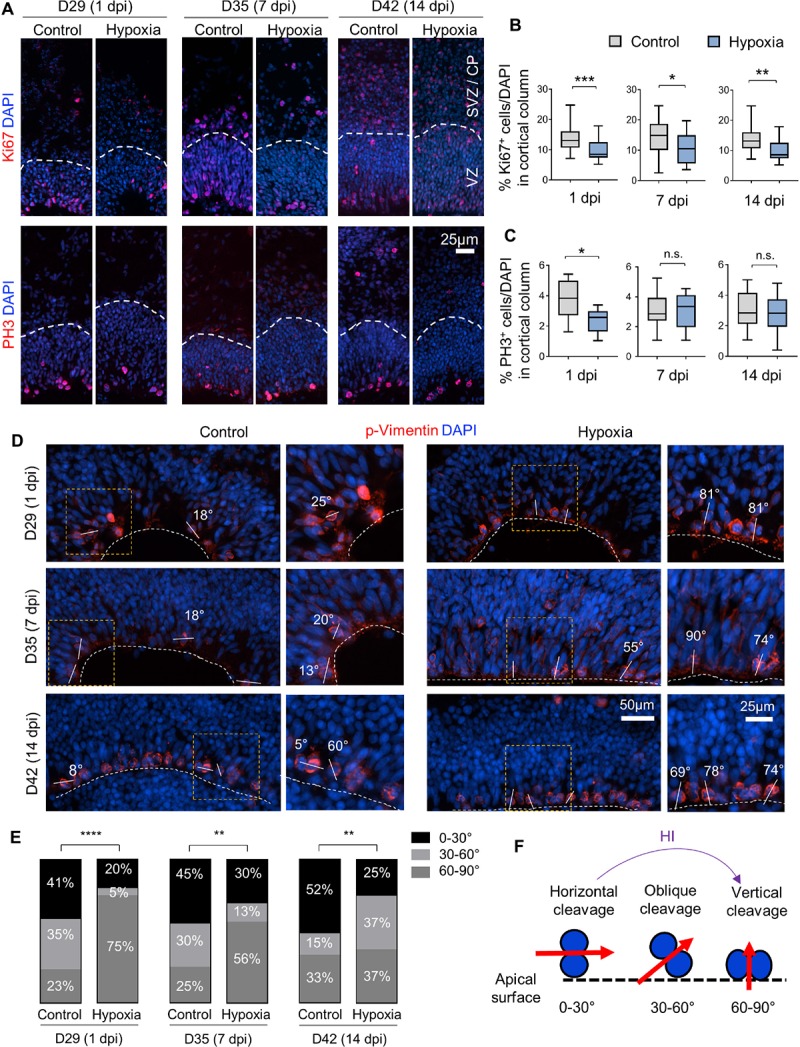
Hypoxic injury reduces proliferation and induces a shift of cell division mode in cerebral organoids. **(A)** IF images for Ki67 and G2/M mitotic marker PH3. White dashed lines delineate VZ and SVZ/CP-like layers in 100 × 300 μm cortical columns. **(B,C)** Quantifications of Ki67^+^ cells **(B)** and PH3^+^ cells **(C)** in cortical columns. Student’s *t* test, *n* = 9 organoids. **(D)** IF images of phospho-Vimentin at apical ventricular surface. Dashed white lines mark the apical surface of ventricle structures. Solid white lines and number labels indicate cleavage plane angle of dividing cells relative to apical surface. High magnification images of boxed area are shown in right panels. **(E)** Quantifications of the prevalence of dividing cells with vertical (defined as 60–90° relative to apical surface), oblique (30–60°), and horizontal (0–30°) cleavage plane at apical ventricular surface of cerebral organoids at indicated time points in control or after HI. Chi-square test, *n* = 20–25 cells quantified per condition. **(F)** Schematic model of a shift of cleavage plane from horizontal to vertical in proliferating cells at apical ventricular surface induced by transient HI. ^*^p < 0.05; ^∗∗^*p* < 0.01; ^∗∗∗^*p* < 0.001; ^*⁣*⁣**^p < 0.0001; n.s., not-statistically significant.

To evaluate the effect of HI on cell proliferation, we first assessed the percentage of cells expressing the proliferation marker Ki67, which showed a significant reduction in cortical columns immediately after HI, but also at 7 or 14 dpi as compared to controls (Figures 4A,B). We next quantified the percentage of cells expressing the G2/M phase marker phospho-histone H3 (PH3), and found that the vast majority of PH3^+^ cells resided at apical surface of VZ in both control and HI conditions, indicating that transient hypoxia did not disturb the interkinetic nuclear migration, i.e., cell bodies of radial glia migrate up from the apical VZ surface during G1 phase, and return back after S phase to undergo mitotic division near the VZ surface. HI, however, caused a significant reduction of PH3^+^ cells at apical surfaces at 1 dpi, but not at 7 or 14 dpi (Figure 4C). Put together, the reduced fractions of Ki67^+^ cells in radial cortical columns, but similar proportions of dividing cells in G2/M phase at apical surfaces of VZ suggest that RG in the VZ niche might have recovered faster from HI-induced proliferation slowdown than other progenitor subtypes in outer layers.

Neural stem cells can expand through symmetric divisions with vertically oriented cleavage planes relative to the apical ventricular surface or undergo neurogenesis through asymmetric divisions with horizontally oriented cleavage planes (Chenn and McConnell, 1995; Mione et al., 1997). To assess whether HI affects cell division mode, we performed IF for the mitotic marker phospho-Vimentin, and measured the cleavage plane angles relative to the apical ventricular surface at different timepoints after HI (Figures 4D,F). Quantification showed an increased incidence of vertical cleavage angles at 1, 7, and 14 days after HI (Figure 4E). In general, horizontal cleavage angles were more prevalent in control organoids, whereas after HI, vertical cleavage angles became more frequent (Figures 4E,F). This change of division mode provides a compensatory mechanism to replenish NSC reserve, but at the expense of neurogenesis, which may further diminish oRG and neuroblast populations.

### Isochronic Cohort Analyses Reveal Sensitive Windows and Vulnerability of Differentiating Population in SVZ/CP to HI

**FIGURE 5:**
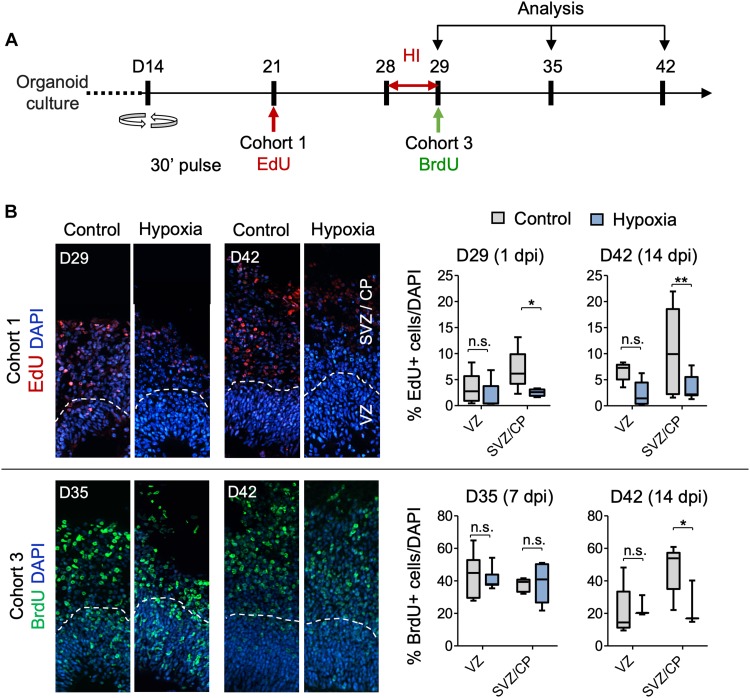
Analysis of isochronic progenitor cohorts reveals vulnerable window and developmental stages affected by HI. **(A)** Timeline of experimental paradigm to analyze different isochronic cohorts (cohort 1 and 3) of neural precursors and their progeny that are birth-dated at different time points relative to transient HI using 30 min EdU or BrdU pulses followed by chase periods until analysis. **(B)** Left: Representative fluorescence images show abundance and spatial distribution of BrdU or EdU LRCs of isochronic cohort 1 or 3 at indicated time points during organoid development in control or after HI. White dashed lines demarcate VZ and SVZ/CP-like layers in 100 × 300 μm radial cortical columns. Right: Quantifications of BrdU^+^ or EdU^+^ LRCs in VZ or SVZ/CP layers. Two-way ANOVA followed by a Tukey *post hoc* test; *n* = 9 independent organoids from three different batches. ^*^p < 0.05; ^∗∗^*p* < 0.01; n.s., not-statistically significant.

To further analyze sensitivity of progenitor populations in different stages of differentiation to HI in the developing cortex, we tracked survival and migration of isochronic progenitor cohorts that were birth-dated at different time points relative to transient HI by timed BrdU or EdU pulse-chase studies. Specifically, cerebral organoids were pulsed with BrdU or EdU for 30 min to label proliferative cells in S phase at D21, 28, 29 or 35. After defined chase periods, the number and spatial location of label-retaining cells (LRCs) were analyzed, informing us on the survival, migration and differentiation of a particular isochronic cohort of neural precursors and their offspring (Figure 5A and Supplementary Figure S4A). It is noteworthy that the abundance of LRCs not only reflects survival but also proliferative history, as fast-dividing cells would dilute EdU or BrdU labels by cell division, leading to reduced number of LRCs.

For cohort 1 cells, birth-dated at D21 by EdU, by the time of HI at D28, a majority of these cells would have already migrated out of VZ and settled in outer layers as committed neuroprogenitors or immature neurons (Figures 5A,B). Indeed, as expected, we found few EdU^+^ cells in VZ by D29 or D42, with no significant differences between control and HI conditions (Figure 5B). In contrast, transient HI resulted in a significantly drop in EdU^+^ LRCs in SVZ/CP at both D29 and D42 as compared to controls (Figure 5B). These data indicated a higher vulnerability of the differentiating populations in the SVZ/CP to HI, echoing the finding that FAM107A^+^ oRGs, TBR2 + IPs, and DCX^+^ neuroblasts/immature neurons appeared more sensitive to HI than SOX2^+^ NSCs in VZ.

Cohort 2 cells were labeled by BrdU at D28, thus representing actively cycling cells in S phase at the time of HI (Supplementary Figure S4A). Our initial assumption was that HI-induced DNA damage may aggravate the replication stress in actively cycling cells, rendering them more vulnerable to apoptosis or delay in neural maturation. Surprisingly, we found no significant differences in the relative number or spatial location for cohort 2 LRCs between hypoxia and control conditions at either D29 or D42. As expected, at D29, a majority of cohort 2 cells were localized in the VZ, and by D42, they had mostly migrated out of VZ and settled in SVZ/CP. We observed no ectopic locations of LRCs from this cohort (Supplementary Figure S4B).

Cohort 3 cells, birth-dated at D29, entered S phase immediately after HI. After 1 week of recovery from HI at D35, we detected no drop in LRCs in either VZ or SVZ/CP layers relative to controls (Figure 5B). However by D42, even though most LRCs for this cohort had successfully migrated to outer layers in both conditions with no ectopia, there was a significant drop of LRCs in SVZ/CP in HI group relative to the control cohort during the second week of recovery (Figure 5B). Judged from the developmental timeline, this indicated that the differentiating population in SVZ/CP appeared more sensitive to HI, again echoing our finding of higher vulnerability of the differentiating populations (FAM107^+^, TBR2^+^, and DCX^+^) to HI. Of note, the reduced number of LRCs for cohort 3 in SVZ/CP may also stem from a shift of cell division mode from neurogenesis to self-renewal.

Cohort 4 cells were birth-dated at D35, after 1 week of recovery from HI (Supplementary Figure S4A). When analyzed immediately after EdU-pulse, we found that in both conditions, proliferating cells were largely detected in the VZ, but slightly away from the apical ventricular surface, consistent with interkinetic nuclear migration of RG cells, while a smaller fraction was detected in outer layers (Supplementary Figure S4B). By D42, LRCs for cohort 4 had mostly migrated out of VZ and settled in outer layers in both conditions, with no significant differences in survival or migration patterns (Supplementary Figure S4B).

**FIGURE 6:**
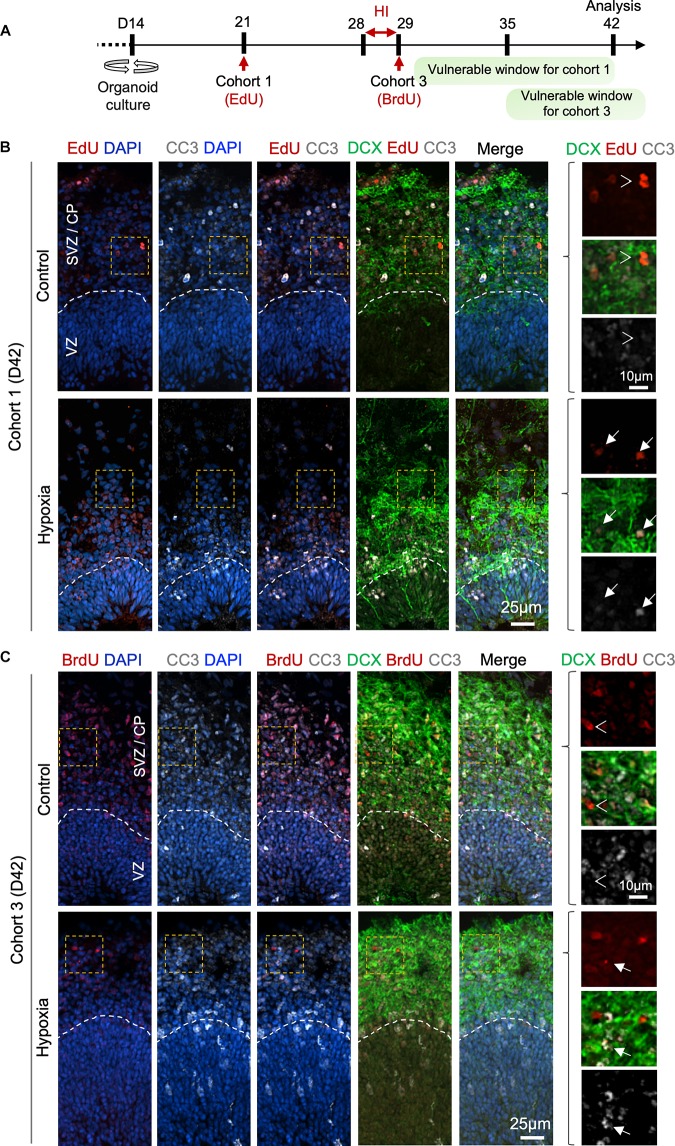
Prolonged effect of transient HI on survival of differentiating population in SVZ. **(A)** Timeline of experimental paradigm to analyze isochronic cohort 1 and 3, birth-dated at D21 or D29 by 30 min EdU or BrdU pulse, respectively, and analyzed at D42 (14 dpi). The vulnerable windows for each cohort are depicted as green bars. **(B,C)** Representative IF images for CC3 and DCX in D42 cerebral organoids. Dashed white lines demarcate VZ and SVZ/CP layers. **(B)** For cohort 1 cells birth-dated at D21 (1 week before HI) and analyzed at D42 (14 dpi), colocalization of EdU, CC3, and DCX staining was observed in HI condition (white arrows), but rarely in control (white arrowheads point to no overlap). **(C)** For cohort 3 cells birth-dated at D29 and analyzed at D42 (14 dpi), no colocalization of BrdU, CC3 and DCX staining was observed in control (white arrowheads), but after HI, apoptotic LRCs with remnant of colocalized DCX and BrdU immunosignals were observed (white arrows). Enlarged images of area outlined by dashed orange box are shown on the right.

To further characterize the differentiation stage during which cohort 1 and cohort 3 cells suffered from HI, we co-labeled LRCs for DCX and apoptotic marker CC3 at D42 (Figure 6A). In regard to cohort 1, we identified in control organoids many EdU^+^ cells in SVZ/CP that were positive for DCX, but few were positive for CC3 label; whereas in HI organoids, we found fewer EdU^+^ LRCs, but they frequently co-labeled for both CC3 and DCX (Figure 6B). For cohort 3, we observed that in control organoids at D42, BrdU^+^ LRCs were largely localized in SVZ/CP, with frequent overlap with DCX, but rarely with CC3. In HI organoids, as expected, fewer BrdU^+^ cells were detected in SVZ/CP, but we found many examples of apoptotic cells exhibiting fragments of BrdU and DCX labels (Figure 6C). Of note, by D42, apoptotic cells induced by HI would have largely been cleared, thus our analysis might have underestimated the extent of overlap of LRCs with CC3. Also notable is the presence of apoptotic cells even in control organoids, congruent with earlier studies (Qian et al., 2016), but the type of cells undergoing apoptosis awaits further characterization in future.

## Discussion

Here, we implemented a transient HI model to study distinct cellular behaviors and coping mechanisms of human neuroprogenitor subtypes in response to hypoxia in human cerebral organoids corresponding to early mid-gestation human brain development. We identified high vulnerability of oRG progenitors but relative resilience of NSCs in VZ niche. We also found a compensatory mechanism to replenish the stem cell reserve after HI through a shift of cell division mode. Furthermore, we defined a critical peri-hypoxia window during which the differentiating neural populations are particularly affected by HI (Figure 6A).

Insights into the pathogenesis of prenatal hypoxia have so far been gathered mostly from animal models or limited analyses of postmortem fetal brains. Conventional *in vitro* cultures lack the complexity of 3D cytoarchitecture, thus unsuitable to address cellular distinction of different progenitor subtypes in response to HI. Cerebral organoids provide an alternative platform to study human brain development and model CNS disorders such as microcephaly (Lancaster et al., 2013; Li et al., 2017), autism spectrum disorders (Forsberg et al., 2018) or Zika virus infection (Qian et al., 2016; Watanabe et al., 2017), among others. In our recent study, we demonstrated engraftment and vascularization of human cerebral organoids upon transplantation into mouse host brains, which facilitates the study of human neurodevelopment in the context of vascularization *in vivo* (Daviaud et al., 2018).

Here, we implemented a transient HI model in cerebral organoids with predominantly dorsal forebrain regional specification, which represent a good approximation of early stages of cerebral cortex development *in vivo* (Kadoshima et al., 2013; Lancaster et al., 2013; Lancaster and Knoblich, 2014; Bershteyn et al., 2017). Human cerebral organoids follow a similar developmental progression as human brains from GW10–18 (Camp et al., 2015; Luo et al., 2016). Based on the localization of FAM107A^+^ oRGs in cortical layers (Pollen et al., 2015), we estimated that our D42 cerebral organoids corresponded to early midgestation human fetal cortex. To ensure reproducibility, we focused our analyses on cortex-like structures close to the surface of organoids.

We found decreased neuroprogenitor proliferation at 1, 7, and 14 dpi, indicating that cell division is an energy demanding process that cannot be met by anaerobic glycolysis alone (De Filippis and Delia, 2011). Different neural cell types at different stages of differentiation fared differently after transient HI. We found a particular vulnerability of oRGs to HI, which highlights the advantage of our species-specific cellular model systems. In comparison, SOX2^+^ neural stem cell reserve in the germinal zone remained stable in size, perhaps reflecting intrinsic resilience to HI, better niche protection, and a compensatory mechanism from a shift of the cleavage plane angles favoring self-expansion. However, the change of division mode would further compromise the oRG and neuroblast populations. In the developing human cortex, the oSVZ is greatly expanded (Smart et al., 2002), and oRG progenitors in the oSVZ contribute to upper-layer neurogenesis as a main driver of the evolutionary expansion of human cortical size (Lukaszewicz et al., 2005; Lewitus et al., 2013). A single oRG progenitor can produce hundreds of deep and upper cortical layer neurons (Pollen et al., 2015). Thus, the demise of oRG progenitors and neuroblasts after HI may ultimately result in reduction of cortical neurons, recapitulating clinically relevant scenarios where cortical gray matter is typically reduced in size in individuals who suffered fetal hypoxia. The current study analyzed up to 14 dpi to avoid confounding factors from increased cell death in organoids after prolonged cultures. Long-term effects of diminished FAM107^+^, TBR2^+^, and DCX^+^ cells on neurogenesis and formation of cortical circuitry await future study.

Our data on change of division mode in neural precursor cells after HI are consistent with earlier studies in neurospheres isolated from P6 rat brains after perinatal hypoxia/ischemia injury, which showed that neural precursors exhibited more frequent symmetric division leading to self-expansion (Felling et al., 2006). Similarly, in adult rat, there is a shift from asymmetric to symmetric cell divisions of SVZ cells during recovery from ischemia *in vivo* (Zhang et al., 2004). The distinct vulnerability of progenitor subtypes to HI found in our organoid model is also in congruence with earlier studies in animal models: during early stage of recovery in a perinatal hypoxia model in P6 rat, Nestin^+^ NSCs in the medial region of dorsolateral SVZ were resilient, whereas the PSA-NCAM^+^ migrating progenitors in the lateral region of dorsolateral SVZ were vulnerable to HI (Romanko et al., 2004). In a neonatal mouse model, mild-to-moderate HI results in reduced cell numbers of NeuroD1^+^ neuroblasts and DCX^+^ neuroblasts/immature neurons, but SOX2^+^ NSC or TBR2^+^ IP populations remained stable (Kwak et al., 2015). A recent study of transient HI model in cerebral organoids also reported resilience of PAX6^+^ radial glia in proliferative zone, but vulnerability of TBR2^+^ IPs in SVZ (Pasca et al., 2019). However, there are important differences between that study and our current study in regard to differentiation protocol, HI paradigm, developmental stages, duration of analysis, and quantification methods, which warrant careful comparison. For instance, dual SMAD inhibitors, as well as BDNF and NT3 were used in the study of Pasca et al. (2019) but not in our study; Matrigel embedding and rotational culture condition were used in our study to enhance oxygen and nutrient exchange in organoid interior, but not in that study; and 3% O_2_ for 24 h was applied in our study vs. 1% O_2_ for 48 h in Pasca et al. (2019). Importantly, we analyzed not only immediate, but also long-term impact of HI, which revealed vulnerability of oRGs and DCX^+^ cells to HI, but only at 7 and 14 dpi, which would have been missed in short term studies. Additional novelty of our study includes: (i) demonstration of massive cell death and cytoarchitectural collapse in developing organoids from continuous hypoxia as compared to transient hypoxia; (ii) unveiling a shift of division mode for radial glia at apical surface of VZ, thereby providing mechanistic understanding of how the NSC reserve might be maintained after HI; (iii) extensive timed EdU or BrdU pulse-chase studies to track different cohorts of neuroprogenitor populations over extended periods, which provided a dynamic picture and delineated cohort 1 and 3 as particularly affected by HI, as well as gave insights into the vulnerable windows.

The distinct responses to HI in different neural cell types may stem from intrinsic differences in handling hypoxic stress or from diverse extrinsic niche environments. Intrinsically, different progenitor subtypes have diverse metabolic needs and distinct transcriptional states. Single cell transcriptomic studies revealed distinct pathways selectively expressed in oRG but not in ventricular RG (vRG) (Hansen et al., 2010). A recent study of HI in cerebral organoids also revealed distinct changes in unfolded protein response pathway occurring after hypoxia (Pasca et al., 2019). Whether molecular distinction accounts for different stress coping and DNA repair capabilities awaits future studies. Extrinsically, different progenitor subtypes reside in unique niches defined by anatomical location, availability of growth factors, cell morphology, and cell behavior (Fietz et al., 2010).

Our isochronic cohort studies revealed that cells birth-dated a week before HI (cohort 1) and immediately after HI (cohort 3) suffered larger losses, specifically in SVZ/CP, coinciding with the timeframe when they are engaged in an active process of differentiation and migration. Unexpectedly, cells that are cycling during the HI (cohort 2) survived well and progressed through normal development despite high replication stress and energy expenditure of cell division. This may reflect intrinsic resilience but also a protective niche in VZ. Indeed, NSCs greatly rely upon anaerobic respiration; they are equipped with abundant glycogen granules, serving as energy substrate during HI; and they have higher level of anti-apoptotic Bcl-2 and Bcl-X_*L*_, endowing them with resistance to cell death stimuli (Romanko et al., 2004). Future transcriptomic profiling of these cohorts will reveal additional molecular basis for their different coping capability against HI.

Here, we focused our study on HI, which can occur due to inadequate oxygen level in maternal circulation due to cardiopulmonary problems or maternal smoking, but our model also provides a versatile platform to study the effects of other environmental insults, such as cerebral ischemia when disruption of blood flow causes deprivation of both oxygen and glucose. We verified hypoxia in our organoids by the stabilization of HIF-1α when exposed to 3% oxygen, while Pasca et al. (2019) also directly measured the partial pressure of oxygen (*p*O_2_) in cortical spheroids using a fiber-optic oxygen microsensor. As both studies used similar gas-controlled chambers with precise calibration, similar profiles of oxygen tension might be expected.

Our experiments were performed with a single hESC line, leaving reproducibility across different stem cell lines unknown at this point. However, it has been recently reported that cerebral organoids derived from different stem cell lines show consistent reproducibility in neuronal cell types (Velasco et al., 2019). Pasca et al. (2019) described reproducible findings using three iPSC lines. Likewise, in the current study, for each experimental condition, at least 2–3 different organoids from three independent batches were used, and we observed high reproducibility.

Our model also open doors for studies in other region-specific cerebral organoids, e.g., organoids with features of hippocampal or cerebellar cytoarchitecture (Muguruma et al., 2015; Sakaguchi et al., 2015). Cerebral organoids can also be cultured for an extended time period to model later stages of corticogenesis. For instance, 2.5-month old organoids have been shown to transcriptionally map to mid-fetal prenatal brain (19–24 post-conception weeks) (Pasca et al., 2015). Hence, despite notable limitations such as lack of vascularization, absence of immune system, and relative early stage of cortical development, cerebral organoids represent an economic and reproducible experimental paradigm to model prenatal HI during human brain development and to develop therapeutic strategies.

In conclusion, our studies established a novel transient hypoxia model in human cerebral organoids that elucidates HI-associated cerebral dysgenesis and provides a starting point for exploring therapeutic options to protect and replenish the vulnerable progenitor population during the critical window.

## Data Availability

All datasets generated for this study are included in the manuscript and/or the Supplementary Files.

## Author Contributions

ND, HZ, and RF conceived and designed the analysis. ND collected the data, performed the analysis and wrote the first draft of the manuscript. CC contributed to data analysis tools. All authors contributed to manuscript revision, read and approved the submitted version.

## Conflict of Interest Statement

The authors declare that the research was conducted in the absence of any commercial or financial relationships that could be construed as a potential conflict of interest.
