# Supplementary material for: Distinct Vulnerability and Resilience of Human Neuroprogenitor Subtypes in Cerebral Organoid Model of Prenatal Hypoxic Injury
Source: Front Cell Neurosci. 2019 Jul 30;13:336. doi: 10.3389/fncel.2019.00336 (PMC6682705; doi:10.3389/fncel.2019.00336)
Supplement: Supplementary file 1 [file Data_Sheet_1.docx]

**Distinct vulnerability and resilience of human neuroprogenitor subtypes in cerebral organoid model of prenatal hypoxic injury**

Nicolas Daviaud ^1,4^, Clement Chevalier ^3^, Roland H. Friedel ^1^, Hongyan Zou ^1,2,#^

**Supplemental Material and Methods**

*Culture of H9 hES cells*

Human pluripotent embryonic stem cells (ESC) were provided by WiCell (H9 hES cells, WAe009-A). For hES cell culture, 6 well-plates were coated with diluted Matrigel (growth factor reduced) (1:100, BD Biosciences) for 20 min at 37°C, and cells were plated and cultured in mTeSR1 media (Stemcell Technologies) supplemented with 2 µM ROCK inhibitor Thiazovivin (Millipore) for 24 hr. Culture media was changed every day until ready for passage or harvest in mTESR1 media (without ROCK inhibitor).

*Cerebral organoid generation*

Human cerebral organoids were generated as described (Lancaster et al., 2013; Lancaster and Knoblich, 2014; Daviaud et al., 2018), with minor modifications: human ES cells were detached using 0.5 mM EDTA (Thermo Fisher Scientific) and plated in round bottom ultra-low attachment 96-well plates (CLS7007, Corning) at a density of 9,000 cells per well in mTESR1 media (STEMCELL Technologies) supplemented with 1% antibiotics (Penicillin Streptomycin, Gibco) for a total of 6 days. During the first 4 days of the culture, media was supplemented with 10 µM of Thiazovivin. Half of the media was changed every day. After 6 days of culture or when embryonic bodies (EBs) reached ~500-600 μm in diameter and when surface tissue began to brighten and formed smooth edges, media was switched to neural induction media (Stemdiff, Stemcell Technologies). Half of the media was changed every day for 3-4 days. After neuroepithelium emerged (usually at ~ day 9-10), organoids were embedded in Matrigel droplets (25 µl, BD Biosciences) and cultured in 6 cm Petri dishes (Falcon) for 4 days in cerebral organoid differentiation media consisting of 1:1 DMEM-F12 and Neurobasal media (Gibco), with addition of 0.5% N2 supplement (Life Technologies), 0.5% ml MEM-NEAA (Gibco), 1% Glutamax (Gibco), 1% B27 supplement without Vitamin A (Life Technologies), 0.1 µM of 2-Mercaptoethanol (Millipore), 2.6 µg/ml Insulin (Sigma Aldrich), and 1% Pen/Strep antibiotics (Gibco). After 4 days, the organoid Matrigel droplets were cultured with addition of Vitamin A on an orbital shaker (VWR) at 85 rpm for 4-6 additional weeks, and then used for experiments (Fig. S1A).

*Histological analyses*

At the indicated time points after HI, organoids were ﬁxed with 4% PFA in PBS at 4°C for 15 min, cryoprotected for 48 hours in 30% sucrose, and then embedded in O.C.T. compound (Tissue-plus, Fisher Healthcare) and stored at -80°C until sectioning into 14 µm sections with a cryostat (CM1850, Leica).

For immunofluorescence staining, non-specific binding sites were blocked with 4% BSA in PBS (Fisher Bioreagents), 0.2% Tween (Tween 20, Acros Organics) and 10% normal donkey serum (Jackson Immunoresearch) for 1 hour at RT, and slices were then incubated with the following antibodies diluted in 4% BSA/PBS, 0.2% Tween: rat anti-BrdU (Novus Biologicals, BU1/75, 1:200); rabbit anti-cleaved Caspase 3 (Abcam, ab2302, 1:100) ; rat anti-CTIP2 (Abcam, ab18465, 1:500); guinea pig anti-DCX (Millipore, AB2253, 1:500); rabbit anti-FAM107A (Sigma-Aldrich, HPA055888, 1:100); rabbit anti- HIF-1α (Abcam, ab179483, 1:200); rabbit anti- HIF-1α (Novus Biologicals, NB100-479, 1:200); rabbit anti-Ki67 (Abcam, ab15580, 1:500); mouse anti-PAX6 (Abcam, ab78545, 1:100); mouse anti-p-Histone H2A.X (Ser 139, Santa Cruz, sc-517348, 1:100); rabbit anti-phospho-histone 3 (Cell Signaling, 9701, 1:500); mouse anti-phospho-Vimentin (Abcam, ab22651, 1:200); rabbit anti-SOX2 (Millipore, AB5603, 1:200); rabbit anti-TBR1 (Abcam, ab31940, 1:500); rabbit anti-TBR2 (Abcam, ab23345, 1:500); mouse anti-tubulin β-III (Tuj1, R&D systems, MAB1195, 1:100). Slides were then washed with PBS-Tween 0.1% and detection was performed with Alexa-coupled secondary antibodies (Invitrogen, Jackson ImmunoResearch) and DAPI nuclear counterstain (Invitrogen).

*Labeling of isochronic NPC cohorts*

Organoids were fixed with cold 4% paraformaldehyde (pH 7.4, Acros Organics) for 12 min and then sectioned on cryostat. EdU staining was revealed using a Click-iT kit (Click-iT EdU Alexa Fluor 594 Imaging Kit, ThermoFisher) and slides were then prepared for immunoﬂuorescence analysis with primary antibodies following standard immunoﬂuorescence procedures. For BrdU analysis, slides were first stained following standard immunoﬂuorescence procedures. Then, a post-ﬁxation was performed for 30 min with 4% PFA/PBS at 4°C and slides were then incubated for 30 min in 2N HCl at 37°C, washed and incubated with anti-BrdU antibody (rat, Thermo Scientiﬁc MA182718, 1:400) overnight at 4°C. After washes, slides were incubated with a secondary antibody for 1 hr at RT and nuclear counterstaining was performed with DAPI.

**Supplemental References**

Daviaud, N., Friedel, R.H., and Zou, H. (2018). Vascularization and Engraftment of Transplanted Human Cerebral Organoids in Mouse Cortex. *eNeuro* 5(6). doi: 10.1523/ENEURO.0219-18.2018.

Lancaster, M.A., and Knoblich, J.A. (2014). Generation of cerebral organoids from human pluripotent stem cells. *Nat Protoc* 9(10)**,** 2329-2340. doi: 10.1038/nprot.2014.158.

Lancaster, M.A., Renner, M., Martin, C.A., Wenzel, D., Bicknell, L.S., Hurles, M.E., et al. (2013). Cerebral organoids model human brain development and microcephaly. *Nature* 501(7467)**,** 373-379. doi: 10.1038/nature12517.

**Supplemental Figure Legends**

**Figure S1: Cerebral organoid development**

A) Schematic depiction (top) and phase-contrast images (bottom) of stepwise derivation of cerebral organoids from hESC.

B) Immunofluorescence images of cerebral organoids at D42 demonstrate ventricle-like structure (V) aligned with NSCs and their offspring in various stages of differentiation as shown with respective markers: Ki67 for proliferating cells, SOX2 for neural stem cells, FAM107A for outer radial glia, TBR2 for intermediate progenitor cells, DCX for neuroblasts, and CTIP2 for deep-layer subcortical neurons. DAPI for nuclear counterstaining.

**Figure S2: Continuous hypoxia severely compromises structural integrity and disrupts cerebral organoid development**

A) Timeline of experimental paradigm of subjecting developing cerebral organoids to continuous hypoxia (3% O_2_) from D28 to D42.

B) Representative fluorescence images of DAPI nuclear staining show that continuous exposure to HI for 2 week severely disrupted cortical layers, leading to cytoarchitectural collapse in cerebral organoids. V: ventricle-like structures.

C) Representative immunofluorescence images of D42 cerebral organoids for radial glia stem cell markers SOX2 and PAX6 and neuronal marker β-III tubulin (TUJ1) demonstrate that 2 week-HI resulted in marked reduction of ventricular neural stem cell pool (SOX2+, PAX6+) and near obliteration of cortical neurons (TUJ1+). Representative 100 µm-wide cortical columns at organoid periphery are shown. Dashed white lines demarcate VZ from outer layers of SVZ and CP.

**Figure S3: Preserved structural integrity of cerebral organoids in a transient hypoxia model**

A) Fluorescence images of DAPI nuclear staining of D42 cerebral organoids in control condition or 14 days after transient HI (D28-29). No apparent structural disruptions were detected. V: ventricle-like structures.

B) Representative immunofluorescence images for neuronal marker β-III tubulin (TUJ1) and stem cell marker SOX2 in cerebral organoids at D35 or D42 (7 or 14 dpi) in control condition or after transient HI. No disorganization or ectopic cell migration was observed.

C) Measurement of average thickness of all cortical layers in cortical column combined (top), of VZ (middle), or of SVZ/CP layers (bottom) after indicated periods of recovery after HI or control. No significant differences were detected between conditions. Two-way ANOVA followed by a Tukey post hoc test; n= 9 independent organoids from 3 different batches. n.s., not significant.

**Figure S4: Analysis of isochronic progenitor cohorts affected by HI**

A) Timeline of experimental paradigm to analyze different isochronic cohorts (cohort 2 and 4) of neural precursors and their progeny that are birth-dated at different time points relative to transient HI using 30-minute EdU or BrdU pulses followed by chase periods until analysis.

B) Left: Representative fluorescence images show abundance and spatial distribution of BrdU or EdU LRCs of isochronic cohort 2 or 4 at indicated time points during organoid development in control or after HI. White dashed lines demarcate VZ and SVZ/CP-like layers in 100 x 300µm radial cortical columns. Right: Quantifications of BrdU^+^ or EdU^+^ LRCs in VZ or SVZ/CP layers. Two-way ANOVA followed by a Tukey post hoc test; n= 9 independent organoids from 3 different batches. n.s., not significant.
