# Supplementary figures and images for: Distinct Vulnerability and Resilience of Human Neuroprogenitor Subtypes in Cerebral Organoid Model of Prenatal Hypoxic Injury
Source: Front Cell Neurosci. 2019 Jul 30;13:336. doi: 10.3389/fncel.2019.00336 (PMC6682705; doi:10.3389/fncel.2019.00336)

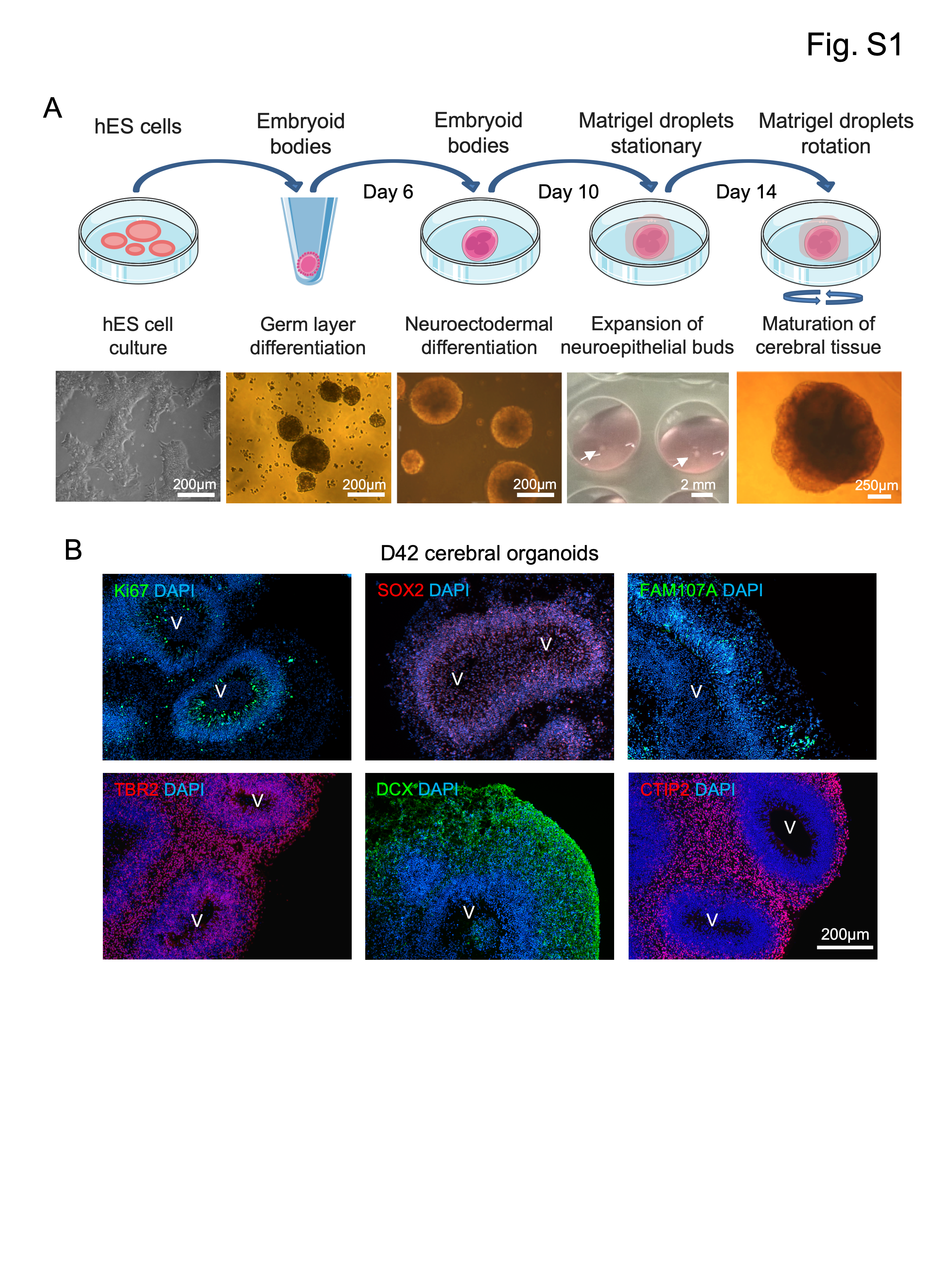

Supplement: Supplementary file 2 [file Image_1.TIF]

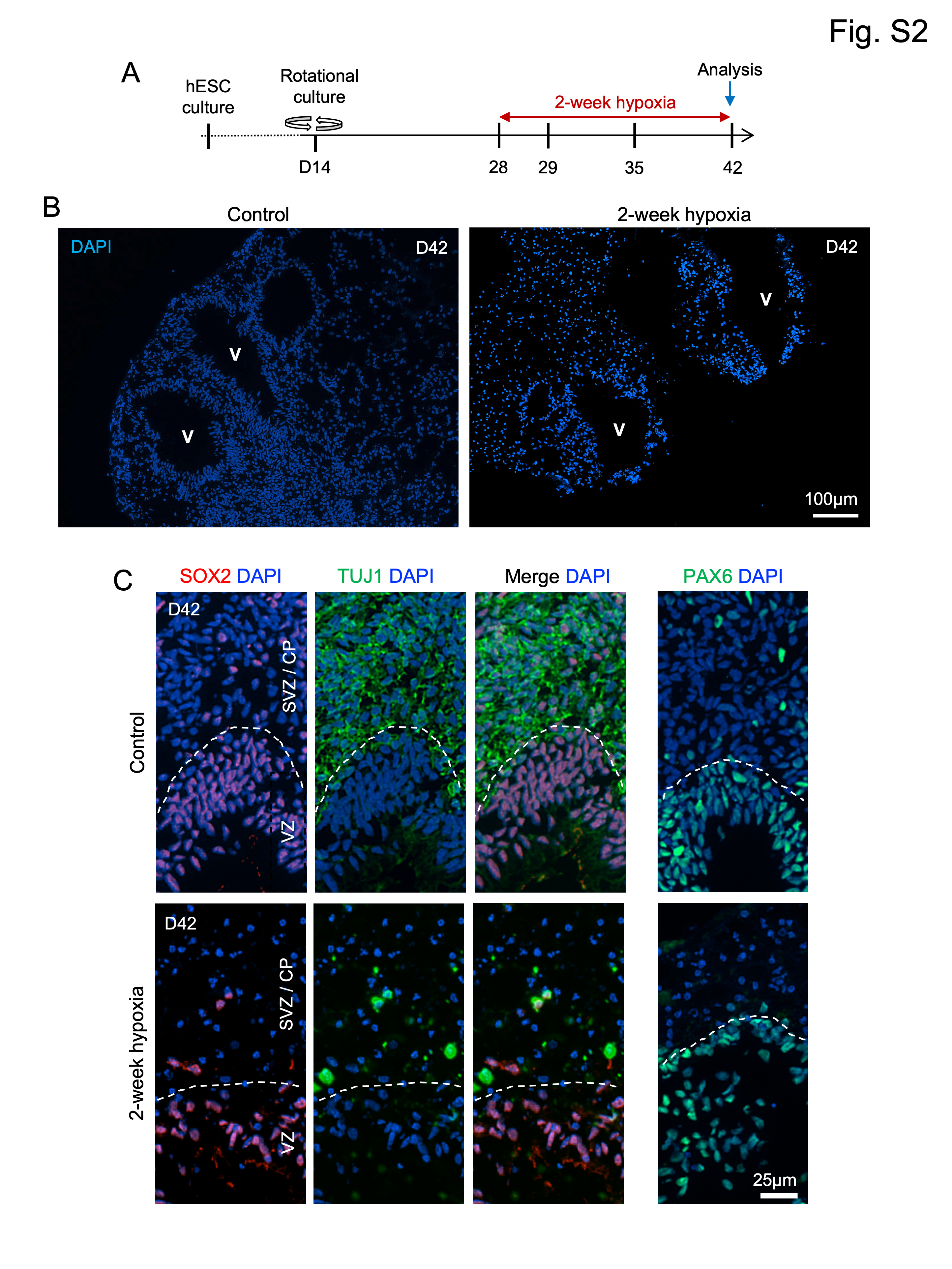

Supplement: Supplementary file 3 [file Image_2.TIF]

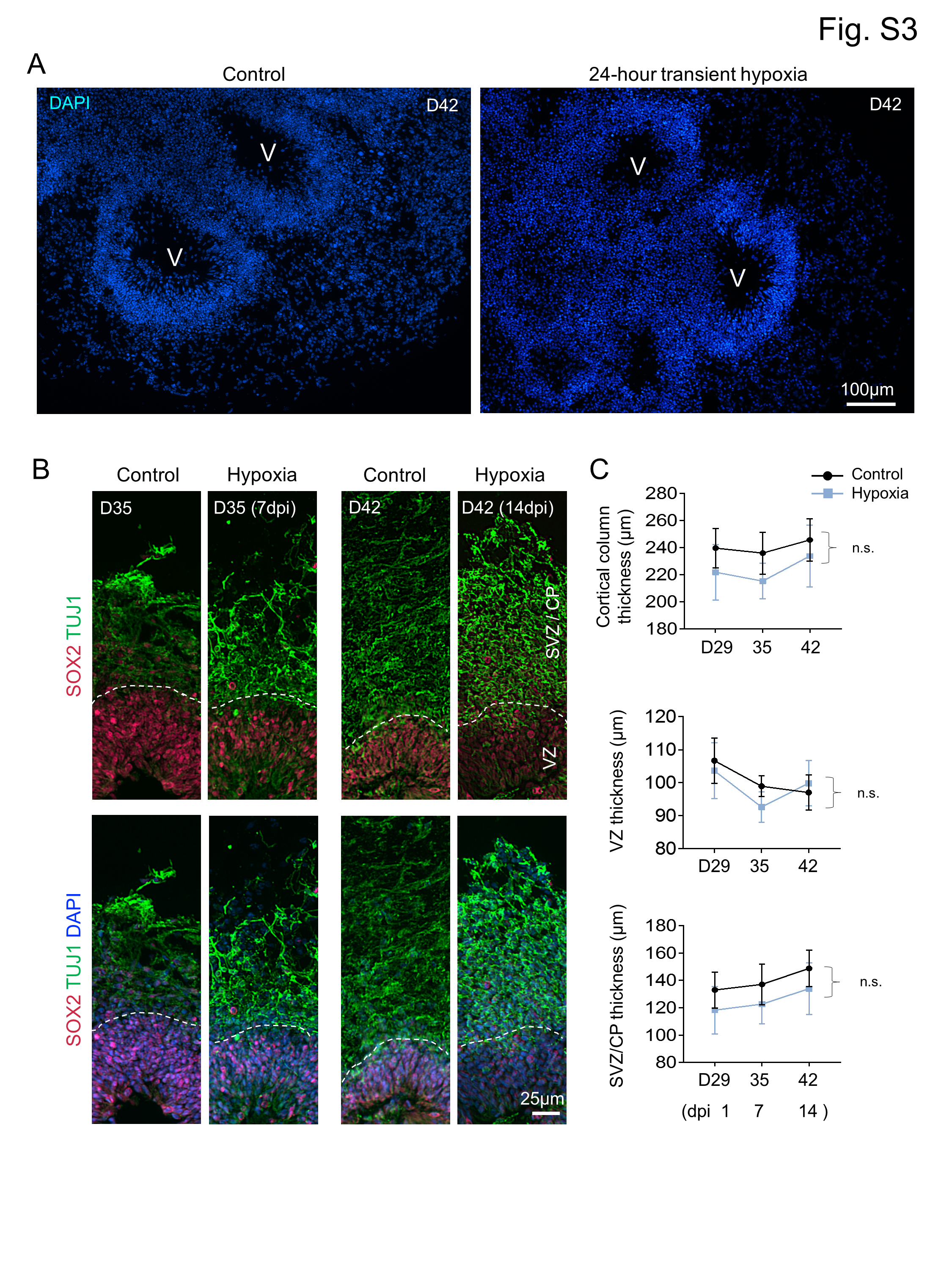

Supplement: Supplementary file 4 [file Image_3.TIF]

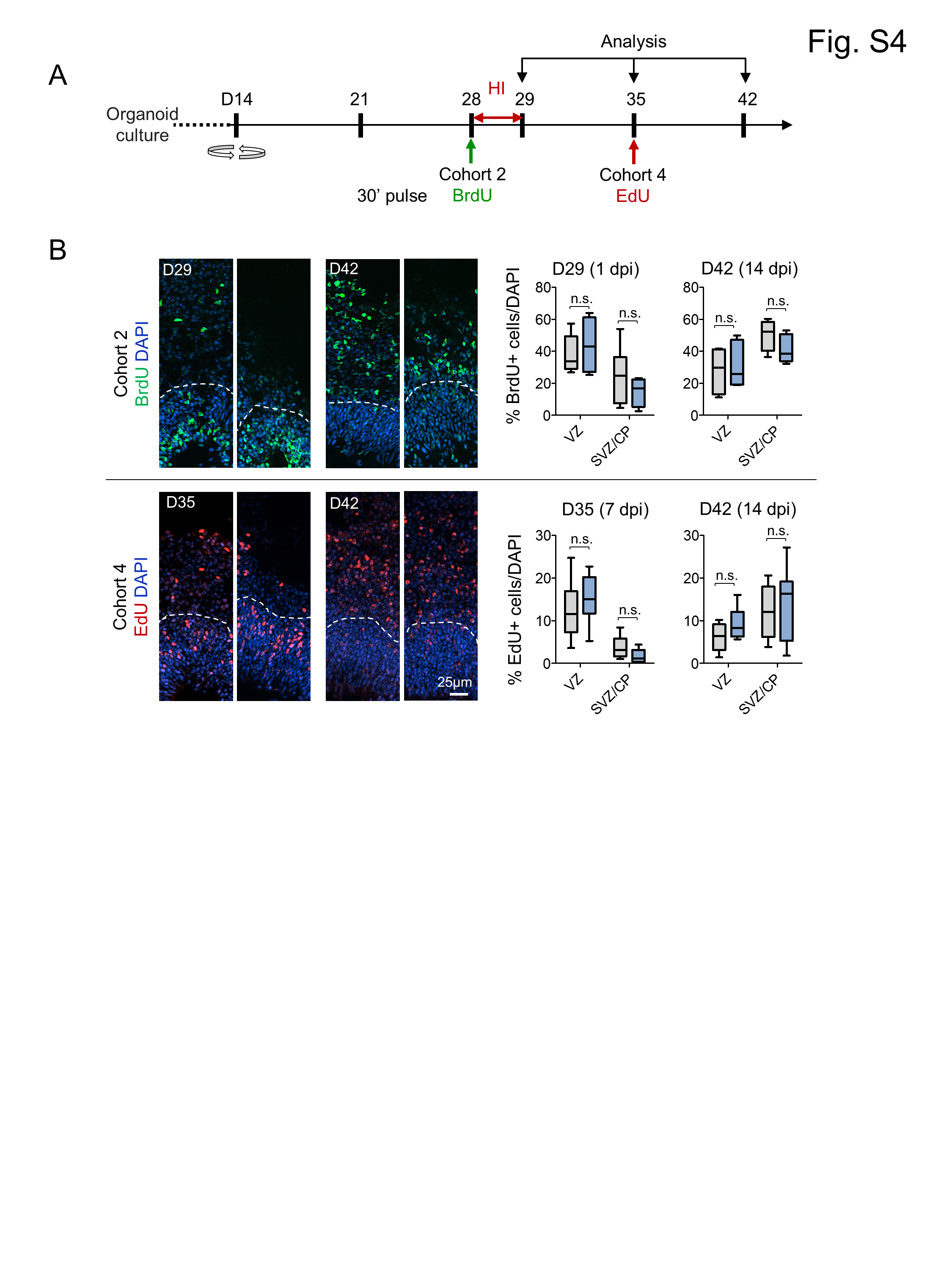

Supplement: Supplementary file 5 [file Image_4.TIF]
